# Sub-10 nm near-field localization by plasmonic metal nanoaperture arrays with ultrashort light pulses

**DOI:** 10.1038/srep17584

**Published:** 2015-12-02

**Authors:** Hongki Lee, Chulhong Kim, Donghyun Kim

**Affiliations:** 1 School of Electrical and Electronic Engineering Yonsei University, Seoul, Republic of Korea, 120-749; 2 Department of Creative IT Engineering Pohang University of Science and Technology (POSTECH), Pohang, Republic of Korea, 790-784

## Abstract

Near-field localization by ultrashort femtosecond light pulses has been investigated using simple geometrical nanoapertures. The apertures employ circular, rhombic, and triangular shapes to localize the distribution of surface plasmon. To understand the geometrical effect on the localization, aperture length and period of the nanoapertures were varied. Aperture length was shown to affect the performance more than aperture period due mainly to intra-aperture coupling of near-fields. Triangular apertures provided the strongest spatial localization below 10 nm in size as well as the highest enhancement of field intensity by more than 7000 times compared to the incident light pulse. Use of ultrashort pulses was found to allow much stronger light localization than with continuous-wave light. The results can be used for super-localization sensing and imaging applications where spatially localized fields can break through the limits in achieving improved sensitivity and resolution.

Localization of near-fields has recently drawn tremendous interests because of the possibility of significantly amplifying light intensity in an extremely small volume^1^. A large part of these interests rely on the excitation and localization of surface plasmon (SP) near metallic nanostructures. Various structures including nanopillars^2^, C-shapes^3^,^4^,^5^, H-shapes^5^, L-shapes^6^, and I-shapes^7^ have been used to localize light fields. Simple nanostructures were evaluated and found to localize near-fields within a very small volume in the lateral surface^8^. On the other hand, gap-based structures such as bowties are known to decrease the localized volume even further^5^,^9^,^10^,^11^. The SP-based near-field localization has been employed in various applications. For example, localization by random nanoislands has been used for imaging endocytosis of adenovirus and intracellular protein perfusion^12^,^13^, while super-resolved detection was performed with periodic nanoholes for gliding microtubules, intracellular proteins, and live bacteria^14^,^15^. The localization proved to be useful for various biosensing applications such as surface-enhanced Raman spectroscopy^16^ and SP resonance (SPR) biosensors in which colocalization of light fields and target molecular interactions can improve detection sensitivity significantly^17^,^18^,^19^,^20^,^21^.

All these approaches are limited to continuous wave operation of light source. Ultrashort light pulses are typically much stronger in the peak power than continuous-wave light and can also take advantage of extreme spatio-temporal localization either directly or after non-linear modification for diverse measurement techniques such as time or frequency resolved gating experiments. Therefore, field localization by pulsed light has been studied for applications that require very high light power that cannot be produced with continuous wave. Such a study employed tip-enhanced photoemission^22^,^23^,^24^,^25^ and was also conducted on 1D grating^26^,^27^ and random structures^28^, while ultrashort SP polariton (SPP) pulses have been observed on a 2D array of circular nanoholes^29^. Furthermore, temporal dynamics of localized plasmon were numerically studied in lithographically defined nanodot arrays^30^,^31^,^32^. In addition, enhancement in dielectric-coupled nanorods and 2D nanoapertures based on L-shapes and T-shapes as well as spatial distribution of electron dipoles that is formed at nanoapertures were investigated for second harmonic generation (SHG)^33^,^34^,^35^,^36^,^37^,^38^. Most of these studies focus on understanding of the way that energy transfer takes place in the near-field via nonlinear effects when fields are localized.

In this work, we intend to study field localization for applications that utilize linear properties, such as photoacoustic imaging and photothermal stimulus of biological tissue^39^,^40^,^41^. Moreover, we are interested in spatial characteristics, rather than temporal dynamics, which is critical to explore new possibilities in super-resolved imaging and stimulus on a spatial scale that may previously have been unavailable. While spatial control of localized field distribution was attempted by phase shaping of femtosecond light^27^ and tip-enhanced coupling with a metallic nanoparticle^42^, we consider here largely centrosymmetric simple nanostructures to obtain localized near-field in various shapes. For centrosymmetric structures, nonlinear effects are mainly attributed to χ^(3)^ nonlinearity, which is much weaker than χ^(2)^ nonlinearity, i.e., linear effects may dominate nonlinear effects when fields are localized by the centrosymmetric simple structure^43^. More complicated structures that often employ nanogap structures have been used, for example, in near-field scanning optical microscopy^44^, which we have excluded here for reasons including the difficulty in fabrication and reproducibility. Even for the nanostructures that we consider, emphasis has been placed upon the ‘simple’ nature from the perspective of implementation, i.e., aperture designs that may be easily mass-produced while exhibiting strongly localized field have been sought.

## Results and Discussion

### Localized near-field distribution

**Figure 1:**
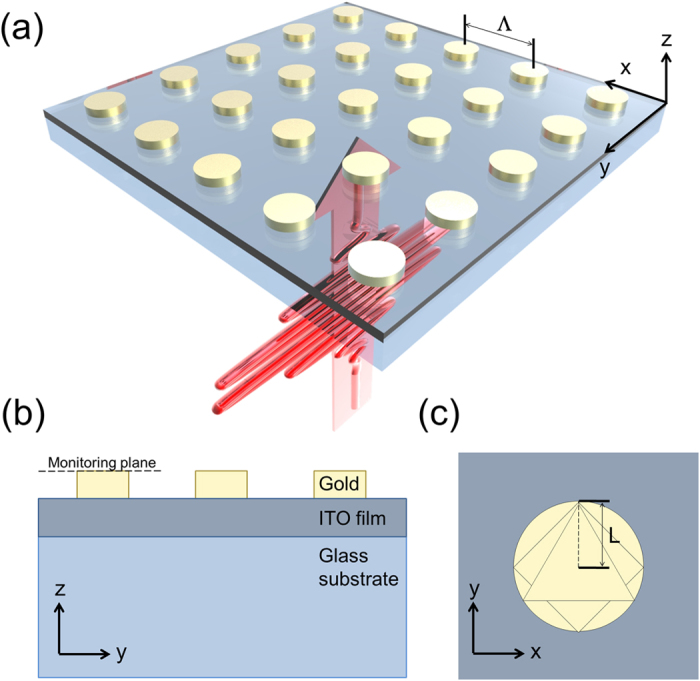
(**a**) Illustration of the schematic model used in the calculation of a gold nanopost. A Gaussian pulse was assumed to be linearly polarized along y-direction at normal incidence. (**b**) The apertures were assumed on an ITO film and a quartz substrate. The fields were measured in the monitoring plane at the top of a nanoaperture. (**c**) Circular, rhombic, and triangular apertures were considered with the aperture length (**L**) in an array of period Λ.

We have considered nanopost apertures of three shapes with which to localize SP distribution and associated near-fields, i.e., circular, triangular, and rhombic apertures as illustrated in Fig. 1. The calculation was performed by varying light wavelengths and geometrical parameters such as period and size which can affect localized near-fields produced on the nanostructures. Details of the calculation appear in Model and methods. For discussion hereafter, the size of an aperture is represented by ‘aperture length’ (L) that refers to the radius for circular apertures and the center-to-vertex distance for triangular and rhombic apertures, as shown in Fig. 1c.

**Figure 2:**
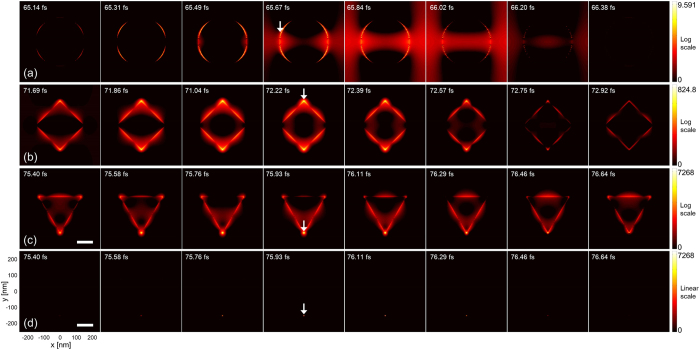
Time-lapse near-field distribution produced by (**a**) circular, (**b**) rhombic, and (**c**) triangular nanoposts. Color bar is in log scale of light intensity. (**d**) Field distribution by triangular nanoposts in linear scale. Arrows represent intensity maxima for each nanopost shape. Calculation was performed with λ_0_ = 850 nm. The pattern period and the aperture length were fixed, respectively, at 500 and 150 nm. The direction of the incident light polarization is along the vertical axis of the images. Scale bar: 100 nm.

Initially, we investigate temporal characteristics of the near-field and then its spatial properties. Figure 2a–c shows temporal evolution of near-field distribution under pulsed light operation when λ_0_ = 850 nm. The pattern period here is fixed at Λ = 500 nm, while the aperture length is identical at L = 150 nm. First of all, it is clear that light pulses induce near-fields to localize spatially. Field localization takes place mainly at vertices of the apertures, which is the manifestation of the Bethe-Bouwkamp model for diffraction by a small aperture in a conducting thin metallic plane^45^,^46^. A primary localized field at much enhanced intensity is marked with an arrow in Fig. 2. Note that the scale of the intensity color bar is logarithmic. Therefore, a side mode or background wave distribution is much weaker in intensity than it appears. For comparison, intensity distribution produced by triangular apertures is also presented on a linear scale in Fig. 2d, which clearly emphasizes the dominance of localized peaks over the background. Note that modeling based on dielectric nanoapertures, metallic nanograting, and thin films indicates that the strong light localization by ultrashort pulses may largely be associated with the plasmonic effect, aided by the relatively minor tip or edge effect that is always present due to the sharp vertices. Apertures produce a symmetric distribution of localized fields along the rim because of the geometrical symmetry and light polarization, although each of the fields is highly localized in shape. For circular apertures in Fig. 2a, localization on two distinct sides is observed on the rim regardless of incident light polarization, which appears in perpendicular to the incident light polarization as a result of the interference of SP modes due to the scattering and reflection by the nanoapertures^47^. A larger nanoaperture may give rise to higher-order plasmon resonance, the effect of which, however, is shown to be relatively limited in Fig. 2a^48^. A rhombic aperture presented in Fig. 2b also shows symmetric intensity peaks at vertices that are apart from each other by the square sides.

**Figure 3:**
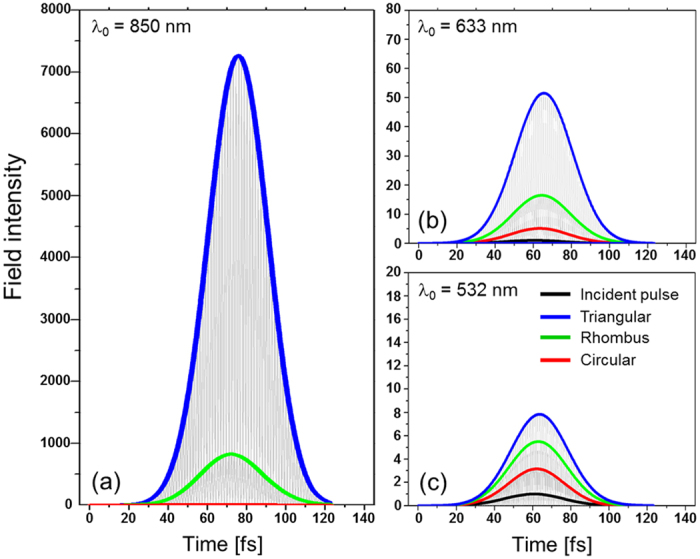
Temporal characteristics of localized field intensity by triangular, rhombic, and circular nanoapertures at different wavelength: (**a**) λ_0_ = 850, (**b**) 633, and (**c**) 532 nm.

Light intensity was observed to oscillate temporally due to the coupling of SPPs at the top and bottom interfaces of nanoposts, as shown in Fig. 3^49^,^50^, where the intensity is normalized by the peak incident light intensity. Figure 3a–c presents the envelope of temporal variation of light intensity at three wavelengths of λ_0_ = 850, 633, and 532 nm for each of the apertures that is measured at the intensity peak position (marked by arrows in Fig. 2). The aperture length and period were again L = 150 and Λ = 500 nm. Each figure presents an incident light pulse as a reference. The intensity variations shown in Fig. 3 contain extremely fast oscillations enveloped with a broad increase of intensity followed by a decrease, which is a result of radiation damping of SP through scattering at nanoposts. In other words, plasmon waves excited at the bottom interface transfer photon flux towards neighboring apertures and increase overall energy density in addition to the pulse directly incident at the bottom^49^. The increased energy is then driven towards the top and the energy density near bottom surface decreases. This process is reversed afterwards, i.e. the energy density transfers from the top to the bottom surface. This periodic energy oscillation is damped and leads to a decrease in the intensity.

Most notably, Fig. 3 demonstrates the observed intensity after localization to be much higher with triangular apertures: at λ_0_ = 850 nm, shown in Fig. 3a, localized fields produced by triangular apertures are stronger by I_Δ/⋄_ = 8.81 (=7268/825) and I_Δ/∘_ = 758 (=7268/9.59) times than those of rhombic and circular apertures, respectively, in terms of the ratio between peak intensities. Higher peak intensity of light with triangular apertures is attributed to light scattering by way of more efficient SP localization, which is in agreement with the results reported previously for continuous-wave light operation^51^,^52^,^53^. The trend of the strongest localization with triangular apertures was consistently observed at other wavelengths, though the peak intensity ratio was reduced to I_Δ/⋄_ = 3.12 and I_Δ/∘_ = 10.1 at λ_0_ = 633 nm (Fig. 3b) and to I_Δ/⋄_ = 1.43 and I_Δ/∘_ = 2.48 at λ_0_ = 532 nm (Fig. 3c). The maximum field intensity produced by each aperture is listed in Table 1. Interestingly, we do not see Rayleigh-like scattering. Rather, Table 1 shows that the peak light field intensity increases with wavelength. The red-shift observed in Table 1 is closely related to retardation effects as the conduction electrons move out of phase and also the absorption process which damps the energy oscillations between top and bottom aperture interfaces^54^,^55^. Absorption in metallic nanoapertures is associated with the radiative scattering loss by the apertures, which contributes to the plasmon damping and thereby significant broadening of the resonance peak, as well as the ohmic loss^49^,^56^. Increased field intensity suggests lower damping, which accompanies excitation and localization of SP, with a longer wavelength.

For imaging applications, presence of a single prominent light field is desired within a diffraction-limited field-of-view^8^, which makes circular apertures undesirable based on the obtained near-field distribution in Fig. 2a. While rhombic and triangular apertures were found to be similar in terms of spatial localization, triangular apertures induce the strongest near-field localization, thereby have been of primary interest in the following discussion.

### Time delay and pulse width

Another important temporal characteristic is that the envelope exhibits a time delay which represents the time for re-emission of SP fields after scattering and is associated with the finite damping of the driven SP resonance^57^. In order to obtain the time delay, the time that corresponds to the peak intensity (*t*_*ref*_) was first calculated in a reference system consisting of buffer without metallic nanostructure. This is subtracted from the peak time *t*_*nano*_ in the presence of nanostructure, i.e., the time delay t_d_ = *t*_*nano*_ − *t*_*ref*_. The relative position of the monitoring plane and the incident light conditions were identical in both cases. At λ_0_ = 850 nm, triangular apertures show the longest delay of t_d_ = 14.49 fs, compared to 10.95 fs and 4.227 fs for rhombic and circular apertures (listed in Table 2). Interestingly, it is observed from Table 1 and 2 that stronger field localization coincides with a longer delay and the time delay increases in general at longer wavelengths. The longest delay is observed for triangular apertures followed by rhombic and circular apertures. The correlation between field localization and time delay appears to be indicative of electron damping process associated with localization of SP as a result of plasmon dephasing time that is directly proportional to the field enhancement and agrees well with the results of earlier studies^58^,^59^,^60^.

Table 3 presents the variation in the pulse width (Δt) which arises from the re-radiation and interference of excited SP waves after scattering and reflection by the nanoapertures of various shapes. Interestingly, the temporal pulse width increases with wavelength. Also worth a note is that the pulse width is reduced for much part of the data compared to the incident pulse width (=35.97 fs) in line with a result obtained for an off-resonant particle plasmon excitation^61^. This may be linked partly to the advancement of the transmitted pulse in time for extremely short pulses^62^. In general, the degree of the pulse width variation is not significant, suggesting that the effect of dispersion may not be dominant, which is in agreement with the results reported in ref. 63.

### Field intensity and time delay for more general aperture geometry

**Figure 4:**
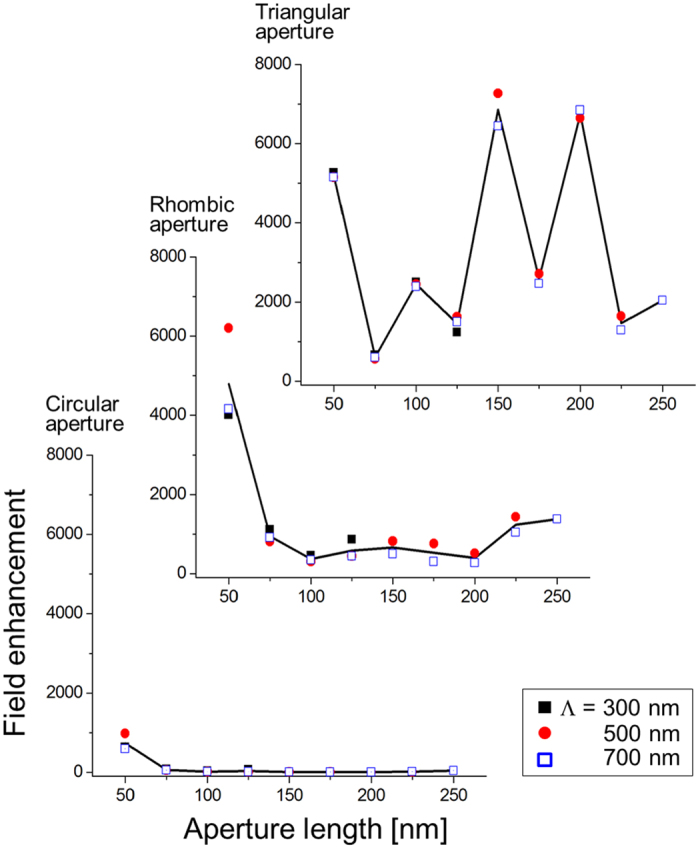
Field enhancement as the aperture length (L) is varied in the range of L = 50 ~ 250 nm at period Λ = 300, 500, and 700 nm for circular, rhombic, and triangular apertures at λ_0_ = 850 nm.

Figure 4 presents the field intensity enhancement obtained with apertures of a range of geometrical parameters including L = 150 nm and Λ = 500 nm, in which several observations are worth a look. Clearly, increased localization from circular to rhombic and triangular apertures enhances localized field strength in general, which is consistent with the results of Fig. 3. While the effect of aperture period is weak, aperture length affects the field strength significantly. The trend appeared consistently in square and circular apertures as well. In Fig. 4, a small aperture gives rise to substantial field enhancement in circular and rhombic apertures due to the plasmonic coupling of near-fields. In fact, the disparity with respect to the aperture shape becomes less noticeable for small apertures, which suggests that the resonant behavior is associated with SP localization within an individual aperture due to ‘intra-aperture coupling’, rather than coupling of localized plasmon between neighboring apertures (‘inter-aperture coupling’) that would stand out with a large aperture at a fixed period. Such a behavior is in good agreement with a report using 2D nanogratings^64^.

**Figure 5:**
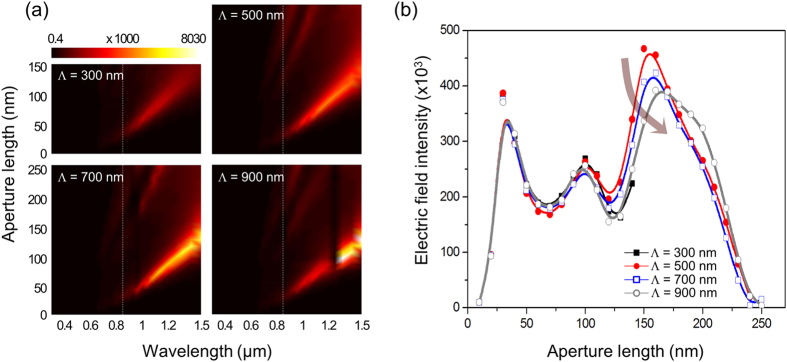
(**a**) Near-field intensity spectra produced by a triangular aperture for 0.3 μm ≤ λ_0_ ≤ 1.5 μm with aperture length (L) and period (Λ) in the range of 0 < L ≤ 250 nm and 300 nm ≤ Λ ≤ 900 nm (Λ in a step of 200 nm). For Λ = 300 nm, 0 < L ≤ 150 nm. (**b**) The field intensity as the aperture length is varied across the dashed lines shown in (**a**) for each period. The arrow represents an increase of aperture period from Λ = 300 to 900 nm. Due to the convenience of calculating impulse response in the *k*-space, the wavelength is fixed at λ_0_ = 843 nm, rather than at 850 nm.

Strong field enhancement in the case of triangular apertures is accompanied by visible fluctuation as the aperture varies in size. For more detailed analysis of the effect of geometrical parameters, full intensity spectra of localized fields with respect to size and period were calculated from the impulse response and by Fourier-transforming the corresponding response of the triangular structure in the frequency domain^65^. The results are presented in Fig. 5a for a wavelength range of 0.3 μm ≤ λ_0_ ≤ 1.5 μm. The intensity was integrated in a 10 × 10 nm^2^ square unit that encloses the top vertex of the triangular aperture at the center and normalized by the incident peak intensity. Because of the normalization process of Fourier transformation, the magnitude of the enhanced field intensity does not coincide with the data presented in Table 1 and Fig. 4. The intensity spectrum, in general, differs from Rayleigh-like scattering as the spectrum represents light scattering and absorption through excitation of localized plasmon modes, where both processes depend on geometrical and material parameters^66^. To the first degree, field intensity tends to increase with aperture size until it reaches a peak at a wavelength in the near-infrared waveband. In other words, the size of an aperture would not affect field intensity monotonically. Figure 5b which shows field intensity variation with aperture length at a fixed wavelength λ_0_ = 843 nm (marked as a dashed line in Fig. 5a) suggests that a resonant behavior should appear in the intensity spectra in line with what was observed in Fig. 4, i.e., much enhanced light scattering is observed when L ≈ 30, 100, and 160 nm. As before, the effect of aperture size is much stronger, whereas that of period is relatively limited. Little dependence on the period is particularly clear when an aperture is much smaller in size than the period due to the nature of intra-aperture coupling. Overall, the degree of intra-aperture coupling vs. inter-aperture coupling depends on geometrical parameters, thereby, causing diffractive resonance. On the other hand, the aperture length that corresponds to the strongest enhancement shifts slightly to a larger value, as the aperture period increases, from L ≈ 150 nm at Λ = 500 nm to L ≈ 160 nm at Λ = 900 nm (an arrow in Fig. 5b used to denote an increase of aperture period from Λ = 300 to 900 nm). The enhancement hints at the inter-aperture plasmonic coupling between neighboring apertures, i.e., more efficient coupling in this case occurs with a larger aperture if the period increases. However, the field intensity itself is reduced with a longer period since a long period weakens inter-aperture coupling.

### Spatial localization

We are particularly interested in the localization of light fields in the spatial domain. For the evaluation of spatial localization, near-field distribution has been integrated over the total calculation time. This presumes that the detector bandwidth is narrower than the bandwidth that femtosecond pulses represent, which is typically satisfied in experimental applications. In effect, this amounts to recording temporal average as a response when detected by a photosensor.

**Figure 6:**
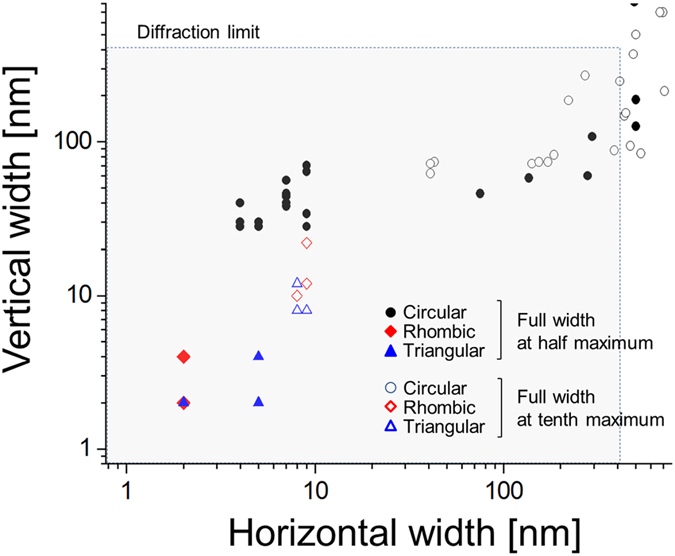
FWHM and full-width-at-tenth-maximum (FWTM) obtained with circular, rhombic, and triangular apertures (L = 50 ~ 250 nm and Λ = 300 ~ 900 nm). The full widths were measured both in the x and y direction. Diffraction limit is presented, assuming use of an objective lens of numerical aperture = 1.

**Figure 7:**
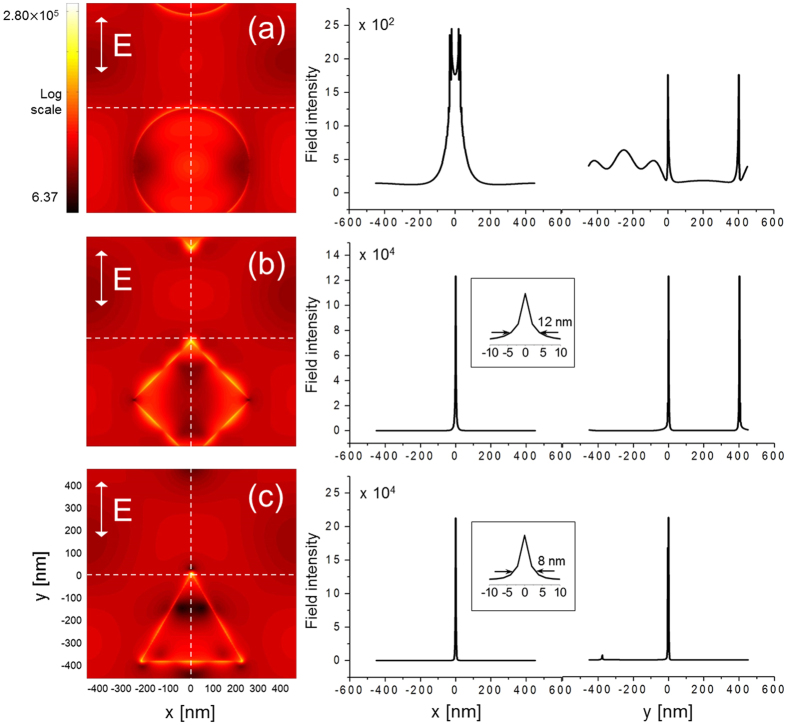
Field intensity distribution obtained with apertures of L = 250 nm and Λ = 900 nm and profiles along the horizontal (x) and vertical (y) direction: (**a**) triangular, (**b**) rhombic, and (**c**) circular aperture. The direction of the incident light polarization is along the vertical axis of the distribution, as shown by the arrow. The main spot is centered in the distribution. The distributions are scaled using an identical color bar. The profiles on the right are measured along the dashed lines in the distribution. Insets in (**b**) and (**c**) show magnified intensity profiles and FWTM along the horizontal direction.

The spatial characteristics of localized fields in terms of full-width-at-half-maximum (FWHM) are presented in Fig. 6 for Λ = 300 ~ 900 nm as the aperture length is varied between L = 50 ~ 250 nm when λ_0_ = 850 nm. To supplement the drawback of FWHM that it does not provide information on the profile, we have also measured full-width-at-tenth-maximum (FWTM). In Fig. 6, it is clear that fields can be localized to be extremely small: with rhombic and triangular apertures, localization below 10 nm in size can be achieved, while it is much less effective with circular apertures. The results imply surprizingly efficient localization of fields, compared to what may typically be observed under continuous wave operation in which case localization below 50 nm was reported to be difficult even if smaller apertures were employed at a shorter light wavelength^8^. Indeed, implementation of such an extreme localization of field was hinted in tip-based experiments using femtosecond pulses^42^. The result may be associated with the complementarity in the space-bandwidth continuum in the sense that the spectrally broad nature of pulsed light source contributes to much stronger localization in the space^67^. Also note that the localization does not take place symmetrically in the lateral plane so that the FWHM changes with respect to the lateral axis, although the ellipticity was in general less significant in rhombic and triangular apertures compared to circular ones. Figure 7 shows the profile of localized fields corresponding to L = 250 nm and Λ = 900 nm. While it presents extreme spatial localization of fields, Fig. 7 also shows that secondary localization modes may exist within a diffraction-limited field-of-view, particularly for rhombic and circular apertures along the vertical direction (y-axis). The secondary mode appears in part to be contributed by the inter-aperture coupling of localized SP, thereby becoming more prominent when aperture length increases compared to the aperture period. One way to circumvent secondary localization is to employ a long period between apertures, although this may deteriorate full-field imaging resolution^68^. Use of a short aperture length may reduce the strength of the secondary modes: if an aperture is too small, the main and the secondary modes may be created together in the diffraction-limited field-of-view, because the location of the secondary modes is largely defined by the aperture structure. In many circumstances, the secondary peak is spatially separated by more than the diffraction-limit from the main peak (see the intensity profile along the vertical direction in Fig. 7b), which makes the existence of the secondary peak irrelevant for super-resolved imaging.

Another aspect to consider is the background field intensity that may potentially affect the signal-to-noise ratio (SNR) in an application. If we define a SNR as the ratio of the main peak intensity to the background, i.e., 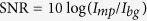 (in dB), where *I*_*mp*_ and *I*_*bg*_ represent the peak intensity of the main mode and the background intensity averaged without main and secondary modes in a diffraction-limited field-of-view, SNR was found to increase with aperture period: in the case of triangular apertures, SNR_min_ = 19.8, 21.8, 23.0, and 23.7 dB for Λ = 300, 500, 700, and 900 nm, respectively. Also, SNR_min_ is higher horizontally (x-direction) than vertically (y-direction). This is because the intensity profile crosses a triangular aperture along the vertical direction and the coupling between plasmon localized at ridges becomes weaker as the period increases, which reduces the average background intensity *I*_*bg*_. SNR can be an indirect measure of field localization and thus suggests well-defined localization of near-field under pulsed light incidence with more than 100:1 intensity ratio with respect to the background.

It is worthwhile to consider the effect of incident pulse duration on the localization. For example, nanosecond pulses allow sufficient time for localized SP distribution to reach equilibrium in the steady state and localized field enhancement tends to be temporally averaged. With longer pulse duration, it is therefore reasonable to suppose that enhanced field intensity would converge on what may be obtained with continuous-wave light incidence. This applies to spatial localization as well, i.e., localized field may broaden as a result of longer pulses.

**Figure 8:**
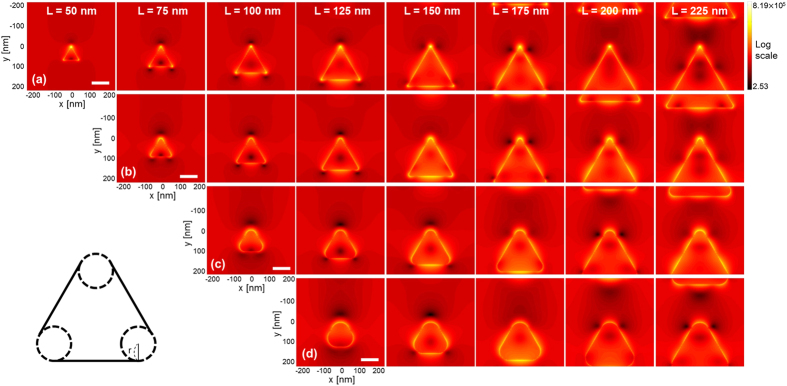
Near-field distribution produced by a triangular nanoaperture with truncated vertices: (**a**) r = 0 (no truncation), (**b**) r = 10 nm, (**c**) r = 30 nm, and (**d**) r = 50 nm. The definition of curvature r is given in the inset (low left). Calculation was performed with λ_0_ = 850 nm. The pattern period was fixed at Λ = 500 nm while aperture length L was varied. The direction of the incident light polarization is along the vertical axis of the images. Scale bar: 100 nm.

**Figure 9:**
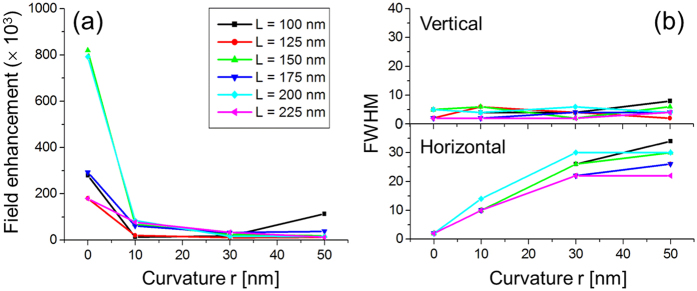
Effects of truncation for triangular nanoapertures: (**a**) field enhancement and (**b**) FWHM (vertical and horizontal). The definition of curvature r is provided in Fig. 8. Calculation was performed with λ_0_ = 850 nm. The pattern period was fixed at Λ = 500 nm while aperture length L was varied.

Note that the fabrication processes will inevitably induce geometry uncertainty. Also, the instantaneous power of ultrashort pulses is so high that the photothermal effect may induce annealing to nanoapertures, which can further modify the geometry and make vertices truncated. For this reason, we have investigated the effects of truncated vertex to make the aperture more practical from an ideal vertex. The results are presented in Fig. 8 which shows near-field distribution of a triangular aperture with truncated vertices. Here, aperture period is fixed at Λ = 500 nm as the aperture length is varied in L = 75–225 nm. The degree of truncation is given by the curvature r defined as in the inset, with r = 10, 30, and 50 nm. It was found that both FWHM and FWTM increase and field enhancement at the peak tends to decrease as a result of increased truncation. An interesting trend to note is that vertical widths along the y direction do not significantly change. In contrast, localized fields tend to significantly broaden along the horizontal direction (x), as r increases. This is clear in Fig. 9. The different trend between horizontal and vertical direction arises from the way that the truncation takes place at the top vertex where field intensity peaks, i.e., the vertical discontinuity across the truncated vertex is almost as abrupt as without truncation. However, the discontinuity is broadened increasingly with r along the horizontal direction.

Regardless of the specifics, we do emphasize that sub-10 nm localization may be obtained in terms of FWHM even after truncation up to r = 10 nm. Considering that the aperture structures studied in Figs 6 and 9 are fairly large on a scale that can be typically fabricated using standard fabrication processes, the results indicate that localization of near-fields on a spatial scale below 10 nm should be implementable under pulsed light incidence and even mass-producible using the nanoapertures. It is suggested that triangular nanoapertures at a long period may produce a well-defined single localized field. If too long, however, image resolution may suffer in case that full-field microscopy is sought based on arrays of localized fields. Therefore, an optimal design of the apertures may ultimately depend on applications.

### Discussion

A very intense pulse that is both temporally localized over a time of about 1 fs and spatially localized within an area of 10-nm diameter as shown in Fig. 2 may have deleterious effects in real applications. Localized beam may vaporize buffer and create bubble within the volume defined by the localized fields and potentially harmful effects may be caused if localized field enhancement is applied to investigating live cells, for example, by the intense energy affecting cell metabolism or even resulting in a rupture of intracellular organelles. Also, possibility of modification of nanoaperture structures due to photothermal annealing may degrade spatial resolution as shown in Figs 8 and 9, e.g., intense temperature gradient may cause metal at the localized field to melt and bring about the flow of molten metal to intensity minima via surface tension driven convection^69^. The short time scale of the localized field may in fact help reduce the effect of thermal diffusion so that such geometrical modification can be minimal. In photoacoustic imaging, the pulse width is shorter than thermal and stress relaxation time and thereby satisfies thermal and stress confinement. In other words, the ultrashort pulses produced by localization would be sufficient to image a 10-nm object^70^.

There are many other challenges if field localization using femtosecond pulses is applied to direct super-localization sensing and imaging, e.g., recording of transient images generated by the ultrashort pulses, far-field detection of near-field, and nanostructure fabrication with uniformity and reproducibility. While the results described here confirm the possibility of spatial localization by ultrashort pulses, direct detection of imaging signals may still remain as a challenge. In this regard, recent emergence of high-speed imaging photosensors is encouraging^71^,^72^. For nanostructure fabrication, novel lithographic techniques including nanoimprint lithography have shown great promise for producing structures of exceptional uniformity in a large area^73^. These new developments are expected to help make the proposed approach a reality in super-resolved detection.

## Concluding remarks

We have explored spatial field localization under ultrashort light pulses based on SP localized by geometrical nanoapertures that are circular, rhombic, and triangular in shape of various combinations of size and period. Triangular apertures were found to produce the strongest spatial localization as well as the highest field enhancement. It is suggested that extreme spatial localization below 10 nm should be achievable, which is significantly smaller than what may be obtained with continuous-wave light. The results can be directly applied to super-localization sensing and imaging that would be difficult to access in continuous-wave operation.

## Model and Methods

### Numerical model

The nanopost apertures were modeled of gold with periods ranging from Λ = 300 to 900 nm in a step of 200 nm. The range of periods precludes near-field inter-aperture coupling as well as degradation of resolution in potential imaging applications^68^. Gold nanoposts were considered with 15 nm height in each of the aperture models on a 30-nm thick ITO layer and a quartz substrate, as shown in Fig. 1b. For circular apertures, the radius was varied from 50 to 250 nm. Triangular and rhombic apertures are equilateral, for which it was the distance between the center and the vertices that varied from 50 to 250 nm. The range of aperture lengths was determined for the ease of fabrication and to avoid reduced field enhancement and excitation of plasmonic quadrupoles^74^. The aperture arrays were assumed to extend to infinity in the lateral plane.

Gaussian pulses at center wavelengths of λ_0_ = 532, 633, and 850 nm were chosen as the light source in the model, although λ_0_ = 850 nm was the main wavelength of interest. The wavelengths were selected for considerations of application such as in photoacoustic imaging^75^,^76^. An incident light beam was assumed to be normally incident on the surface and linearly polarized along y direction. The Gaussian pulse is specified by an envelope function and a sinusoidal carrier and can be expressed as


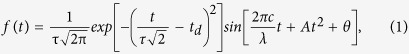


where 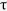 and 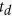 represent the pulse width and the delayed launch time, respectively^77^. The phase constant (*θ*) as well as the chirping coefficient (A) was chosen to be zero. It is thus assumed that the Gaussian pulse exhibits no chirping. In Eq. (1), *τ* was set to be 21 fs so that the temporal FWHM of the Gaussian envelope was 50 fs. The wavelength spectra for λ_0_ = 532, 633, and 850 nm have three different spectral linewidths at 20, 28, and 50 nm. Material refractive indices of gold, ITO, and quartz were taken from refs. 78, 79, 80, respectively.

### Calculation method

Numerical calculation of near-fields was performed with 3D finite difference time domain method with periodic boundary conditions in the lateral dimension. Along the z axis, perfectly matched layer conditions were enforced. The calculation used meshes of a size 2 × 2 × 2 nm^3^ with a total calculation volume defined by one single period of an aperture and an axial depth of 90 nm. To ensure the validity of sub-10 nm near-field localization, calculation was also performed with a smaller mesh size of 1 × 1 × 2 nm^3^ for select cases and results were largely similar without affecting the conclusion. Temporal computation was performed with a time step of 0.004 fs for a total time of 120 fs. Spatial properties were extracted from the near-field integrated over the total time: this is based on the assumption that the characteristic time associated with an application is much longer than the total time that was calculated over. Four monitoring planes that are 10 nm apart in the axial direction (z-axis) were set up, among which the electromagnetic field distribution was measured at the top of a nanoaperture, as shown in Fig. 1b. Temporal characteristics were mainly collected at the intensity peak of the field distribution in the monitoring plane. Post analysis was performed using custom-coded MATLAB.

## Additional Information

**How to cite this article**: Lee, H. *et al*. Sub-10 nm near-field localization by plasmonic metal nanoaperture arrays with ultrashort light pulses. *Sci. Rep*. **5**, 17584; doi: 10.1038/srep17584 (2015).

## Figures and Tables

**Table 1:** Field intensity maximum normalized by the peak intensity of an incident light pulse when fields are localized by various apertures with the aperture length L = 150 nm and the period Λ = 500 nm.

	Circular aperture	Rhombic aperture	Triangular aperture
λ_0_ = 532 nm	3.160	5.499	7.848
λ_0_ = 633 nm	5.125	16.51	51.54
λ_0_ = 850 nm	9.591	824.8	7268

The incident pulse was measured at the ITO surface.

**Table 2:** Time delays (t_d_) when fields are localized by various apertures with the aperture length L = 150 nm and the period Λ = 500 nm, relative to the time that corresponds to the peak intensity in a reference system without metallic nanostructure.

	Circular aperture	Rhombic aperture	Triangular aperture
λ_0_ = 532 nm	0.666	2.052	2.052
λ_0_ = 633 nm	1.848	2.844	4.032
λ_0_ = 850 nm	4.227	10.95	14.49

The relative position of the monitoring plane and the incident light conditions were identical in both cases. Unit in fs.

**Table 3:** Temporal width (Δt) when fields are localized by various apertures with the aperture length L = 150 nm and the period Λ = 500 nm.

	Circular aperture	Rhombic aperture	Triangular aperture
λ_0_ = 532 nm	34.52	35.63	35.19
λ_0_ = 633 nm	35.64	35.78	35.64
λ_0_ = 850 nm	37.18	37.71	35.76

The incident pulse width is 35.97 fs. Unit in fs.
